# OA18 A rare presentation of Polyarteritis Nodosa

**DOI:** 10.1093/rap/rkac066.018

**Published:** 2022-09-28

**Authors:** Shivani Gor, Sara Carty

**Affiliations:** Great Western Hospital, Swindon, United Kingdom; Great Western Hospital, Swindon, United Kingdom

## Abstract

**Introduction/Background:**

Polyarteritis nodosa (PAN) was first described in 1866 as a necrotising vasculitis predominantly involving medium-sized arteries. It is a multiorgan disorder which can cause a range of symptoms from cutaneous ulcers to gastrointestinal haemorrhage. For many patients, the cause remains elusive, however a close relationship has been demonstrated between PAN and hepatitis B infection. We describe a case of a 45-year-old who presented with acute abdomen to the emergency department. She was admitted under the general surgical team and common surgical conditions were ruled out. Imaging later revealed coeliac and splenic pseudoaneurysm.

**Description/Method:**

A 45-year-old woman presented with acute epigastric pain. It was colicky in nature and radiated to the right upper quadrant with some associated vomiting. On examination she looked unwell with a tachycardia but normal blood pressure and temperature. The abdomen was rigid with positive Murphy’s sign. There were no joint or skin anomalies. She was admitted under the surgical team for further investigation.

Bloods tests revealed a raised C-reactive protein (CRP) of 60mg/L. Full blood count, amylase levels, liver and renal function were all normal. Despite antibiotic treatment her clinical condition did not improve. A Computed Tomographic (CT) scan was performed which reported stenosis of the coeliac trunk and dilatation of the coeliac artery. This was accompanied by periarterial inflammatory change. A CT angiogram confirmed a coeliac pseudoaneurysm with evidence of a dissection flap and splenic pseudoaneurysm. Vessel wall thickening was noted, and these changes were in keeping with a vasculitis.

At this point the rheumatology team was involved and further investigations were requested. Anti-Neutrophilic Cytoplasmic Autoantibody (ANCA), Rheumatoid Factor (RF), Anti-Nuclear Antibody (ANA), syphilis and Hepatitis B, C were all negative. Urine dipstick was clear.

A diagnosis of polyarteritis nodosa (PAN) was made and high dose prednisolone was started. She went on to have six cyclophosphamide infusions. Despite immunosuppression and moderate strength analgesia, she had ongoing abdominal pain. Further imaging revealed a thrombus within the coeliac pseudoaneurysm and so treatment-dose low molecular weight heparin was commenced. Her case was also discussed with the hepato-pancreato-biliary surgical team. They confirmed there was no evidence of bowel ischaemia and surgical intervention was deemed too high risk. 6 monthly CT scans to monitor the pseudoaneurysms were advised.

Once the cyclophosphamide infusions were completed, she was started on azathioprine as maintenance therapy. A PET scan was organised which showed no active disease.

**Discussion/Results:**

Given our patients presentation and CT findings, PAN was felt to be the best fit clinical diagnosis. The differential diagnoses to consider here were Takayasu arteritis and mycotic aneurysms. Given our patient’s age, and absence of both clinical and biochemical features of sepsis meant that PAN seemed a more likely explanation.

PAN affects small to medium vessels and causes transmural necrotising inflammation of the arteries. The exact pathogenesis is unclear, but the inflammatory process causes weakening of the arterial wall, leading to aneurysmal dilatation and possible rupture. As a result, the organs perfused by these arteries are at risk of becoming ischaemic.  This weakening of the artery usually occurs at bifurcation points and mostly is seen in the liver or kidney. The overall incidence of aneurysmal rupture is thought to be rare.

Clinical features of PAN vary widely but the most common symptoms are fever, malaise and weight loss. The next most common presentation is an acute peripheral neuropathy or mononeuritis multiplex. Abdominal pain as a presentation of PAN is seen in less than 40% of cases. Typically, the pain comes on after meals due to mesenteric arteritis. Coeliac artery involvement is rare, particularly with the presence of stenosis and thrombosis as in our patient. No definitive management criteria exist but aneurysms bigger than 2cm can be considered for surgery. However, most aneurysms decrease in size with immunosuppression. Angiography is the imaging modality of choice for PAN as it has a sensitivity and specificity of 89% and 90%, respectively.

Initial treatment consists of glucocorticoids and patients with severe disease have improved survival with combination of steroids with cyclophosphamide. Once remission has been attained the steroids can be tapered gradually. Generally, PAN has a low relapse rate but in high-risk patients, maintenance therapy, such as azathioprine, can be introduced.

**Key learning points/Conclusion:**

Although PAN is a well-known form of vasculitis, unusual presentation can occur as in our patient. Abdominal pain is a common symptom resulting in attendances to the emergency department. Our patient was initially thought to have cholecystitis, but she showed no clinical improvement despite standard treatments. A subsequent CT scan then displayed the abnormalities of her coeliac artery. This demonstrates an important lesson of keeping initial differential diagnoses wide and undertaking further investigations until an answer is found. In our patient this was particularly important as she did not display any typical constitutional symptoms of fatigue or fevers usually seen with systemic vasculitides.

Another essential point is, once the diagnosis of PAN has been made and treatment initiated, if the patient continues to complain of symptoms, further imaging should be undertaken. There are two main reasons that this would occur in PAN, the first being rupture of the original aneurysm resulting in end organ ischemia. If this is present urgent surgical intervention may be required. The second is development of thrombosis within the aneurysm, as was the case in our patient.

As with most multisystem diseases a Multidisciplinary Team (MDT) approach is vital. An MDT approach allows prompt diagnosis and initiation of correct treatment which is needed with any systemic vasculitis. In this case, use of a specialist HPB surgical team was crucial in aiding decisions for biopsy, surgical intervention and frequency of surveillance scans.

Lastly, a diagnosis of any chronic rheumatic condition can negatively impact on the patient’s quality of life. Multiple hospital visits, serial scans and the burden of medication can take its toll on patients. Thus, it is good practice to enquire of the patient’s mental health at appointments and decisions around medical care should be shared with the patient.

